# CT-based transformer model for non-invasively predicting the Fuhrman nuclear grade of clear cell renal cell carcinoma

**DOI:** 10.3389/fonc.2022.961779

**Published:** 2022-09-28

**Authors:** Meiyi Yang, Xiaopeng He, Lifeng Xu, Minghui Liu, Jiali Deng, Xuan Cheng, Yi Wei, Qian Li, Shang Wan, Feng Zhang, Lei Wu, Xiaomin Wang, Bin Song, Ming Liu

**Affiliations:** ^1^ Yangtze Delta Region Institute (Quzhou), University of Electronic Science and Technology of China, Quzhou, China; ^2^ School of Computer Science and Engineering, University of Electronic Science and Technology of China, Chengdu, China; ^3^ Department of Radiology, Affiliated Hospital of Southwest Medical University, Luzhou, China; ^4^ Department of Radiology, West China Hospital, Sichuan University, Chengdu, China; ^5^ Quzhou Affiliated Hospital of Wenzhou Medical University, Quzhou People’s Hospital, Quzhou, China

**Keywords:** tumor grading, ensemble learning, clear cell renal cell carcinoma, transformer network, deep learning

## Abstract

**Background:**

Clear cell Renal Cell Carcinoma (ccRCC) is the most common malignant tumor in the urinary system and the predominant subtype of malignant renal tumors with high mortality. Biopsy is the main examination to determine ccRCC grade, but it can lead to unavoidable complications and sampling bias. Therefore, non-invasive technology (e.g., CT examination) for ccRCC grading is attracting more and more attention. However, noise labels on CT images containing multiple grades but only one label make prediction difficult. However, noise labels exist in CT images, which contain multiple grades but only one label, making prediction difficult.

**Aim:**

We proposed a Transformer-based deep learning algorithm with CT images to improve the diagnostic accuracy of grading prediction and to improve the diagnostic accuracy of ccRCC grading.

**Methods:**

We integrate different training models to improve robustness and predict Fuhrman nuclear grade. Then, we conducted experiments on a collected ccRCC dataset containing 759 patients and used average classification accuracy, sensitivity, specificity, and AreaUnderCurve as indicators to evaluate the quality of research. In the comparative experiments, we further performed various current deep learning algorithms to show the advantages of the proposed method. We collected patients with pathologically proven ccRCC diagnosed from April 2010 to December 2018 as the training and internal test dataset, containing 759 patients. We propose a transformer-based network architecture that efficiently employs convolutional neural networks (CNNs) and self-attention mechanisms to extract a persuasive feature automatically. And then, a nonlinear classifier is applied to classify. We integrate different training models to improve the accuracy and robustness of the model. The average classification accuracy, sensitivity, specificity, and area under curve are used as indicators to evaluate the quality of a model.

**Results:**

The mean accuracy, sensitivity, specificity, and Area Under Curve achieved by CNN were 82.3%, 89.4%, 83.2%, and 85.7%, respectively. In contrast, the proposed Transformer-based model obtains a mean accuracy of 87.1% with a sensitivity of 91.3%, a specificity of 85.3%, and an Area Under Curve (AUC) of 90.3%. The integrated model acquires a better performance (86.5% ACC and an AUC of 91.2%).

**Conclusion:**

A transformer-based network performs better than traditional deep learning algorithms in terms of the accuracy of ccRCC prediction. Meanwhile, the transformer has a certain advantage in dealing with noise labels existing in CT images of ccRCC. This method is promising to be applied to other medical tasks (e.g., the grade of neurogliomas and meningiomas).

## 1 Introduction

Renal cell carcinoma (RCC) is the most common kidney tumor (1, 2). Clear cell RCC (ccRCC) is the predominant hypotype of RCC, accounting for about 75-80 (3). With the background of population aging, kidney cancer, especially RCC, keeps rising. When ccRCC progresses to intermediate and advanced stages, lymph node metastasis or distant organ metastasis probably occurs, which leads to dangerous clinical symptoms and a poor prognosis (4, 5). At present, one of the most important pieces of clinical evidence for judging the malignancy degree of ccRCC is given by the Fuhrman grading system, which defines four pathological grades based on nuclear size, shape, staining, and nucleoli (5). Generally, tumors with low invasiveness are classified as grades I–II, and those with high invasiveness are classified as grades III–IV (6).

The preoperative biopsy is the gold standard for evaluating the grade of ccRCC. However, patients undergoing biopsy are at risk of complications, e.g., hematuria (with more than 80% incidence), perirenal hematoma (with about 60%–90% incidence), and infection (7). The procedure of preoperative biopsy is complex and invasive. Besides, biopsy cannot reflect the Fuhrman grade of the whole tumor (8) because of the high spatial and temporal heterogeneity. Thus, preoperative evaluation of ccRCC using a noninvasive procedure for clinical diagnosis is urgently needed. CT examination is the most commonly used non-invasive technique for preoperative diagnosis and follow-up and plays an essential role in diagnosing and treating renal carcinoma, e.g., detection, localization, characterization, and grading of lesions. In some studies, it has been used to evaluate the preoperative ccRCC classification, such as (9–12).

Preoperative noninvasive prediction of ccRCC is conducive to delivering an individualized treatment. Previous studies (12, 13), using radiation characteristics based on multiphase CT, investigated the predictive performance of different machine learning models for discriminating ccRCC. Beyond that (14–17), have shown that convolutional neural networks based on single or multiphase CT images are beneficial for evaluating ccRCC grading. However, the biggest challenge of CT image grading is the existence of noise labels in the image (8). What is a “noise label?” One CT image may contain multiple grades but may have only one label. For example, the grade III–IV grade CT image contains grade I–II tumor areas because the label was obtained from the biopsy of the most severe tumor area of the whole kidney. When encountering noise labels, convolutions uniformly process all tumor regions regardless of their importance, which leads to the inefficiency of classification. CNN makes decisions based on the convolution kernel, which only focuses on the local pixel subset, resulting in the network tending to learn the local mode rather than the global context.

The transformer network, a branch of deep learning, is considered a promising technology for analyzing medical images because it can capture global representations and establish long-distance relationships within the image (18, 19). Therefore, the transformer is suitable for handling CT images with noise labels. We propose a transformer classifier, TransResNet, to predict high-grade ccRCC using CT images. To the best of our knowledge, there have been no investigations about discriminating the low and high nuclear grade ccRCC by combining transformer network and radiological features.

As a result, this research aims to investigate an efficient transformer classifier for predicting the Fuhrman grading of ccRCC based on three-phased CT images.

## 2 Materials and methods

### 2.1 Data preparation

**Patient cohort:** This diagnostic and observational study was approved by the institutional review board (West China Hospital, Sichuan University), and written informed consent was obtained from all patients. Consecutive patients were collected from April 2010 to December 2018 in one hospital. We cleaned the original data according to the following rules: (1) there is no obvious noise in the image of the patient; (2) the patient image has no apparent artifacts. Specifically, all of the pathologically proven ccRCC grades are reconfirmed by experienced radiologists. This work uses the Fuhrman nuclear grading system as the standard grading system. Finally, 759 patients were included in this work.

**CT images:** All patients underwent a multi-slice CT scan with three phases, including unenhanced, arterial, and portal venous phases, using the following systems: LightSpeed VCT (GE Healthcare), Sensation 64 CT (Siemens), or Sensation 16 CT (Siemens). The PCP, CMP, and NP of the MDCT (multidetector CT) examination were acquired for each patient with the following protocol. By using a high-pressure injector at a rate of 3.5 ml/s, 70–100 ml of contrast agents were injected into the antecubital vein. The CMP is the corticomedullary phase contrast-enhanced scan starting 30 s after the contrast agent injection. The NP is the nephrographic phase contrast-enhanced scan starting 90 s after the contrast materials injection. Spiral scanning and thin-slice reconstruction were used for all three phases. The CT scanning parameters of the phases were as follows: the tube voltage was 120 KV; the reconstruction thickness was 1 mm to 5 mm, and the matrix was 512 × 512. All CT scans of patients are converted to color-scale images and reviewed by experienced radiologists in abdominal imaging. The ccRCC images collected from the CMP phase are used in experiments.

**Figure 1:**
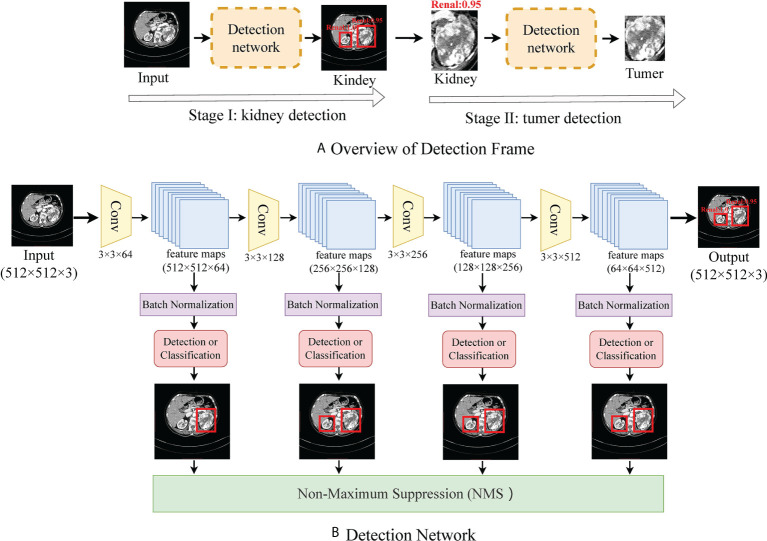
**(A)** shows the flow frame of data processing, in which stage one is the kidney detection network and stage two is a tumor detection network. **(B)** shows the details of the detection network.

**CT Processing:** Medical CT slices are diverse and complex. If CT slices are selected to be the input of the classification model, the results are imperfect and require further optimization. To make a more accurate diagnosis, we preprocess the original CT image by detecting the organ or lesion area from medical scanning. We utilize a tumor detection network to quickly and efficiently obtain the rectangular region of interest of the tumor in each phase image. As shown in **Figure 1**, to reduce the complexity of direct tumor segmentation, the detection frame is divided into two stages: (1) renal organ detection: this detection module is composed of VGG16 (20) without the classification layers (pre-trained on ImageNet (21)), aiming to find the rectangular region of kidneys to mitigate the influence of background of CT scans and reduce the search space of tumors; (2) tumor detection: aims to regress the rectangular region of the tumor accurately and predict the possibility of the tumor.

**Figure 2:**
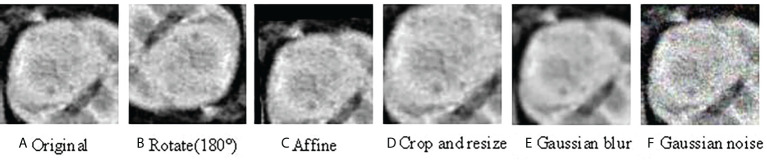
Illustrations of the results using various data augmentation **(A)** shows the original CT image; **(B)** is the CT image after 180° rotation; **(C)** is the CT image after Affine transformation; **(D)** shows the CT image after crop and resize transformation; **(E)** is the CT image with Gaussian blur; **(F)** is the CT image with Gaussian noise.

Because medical data are scarce and difficult to label, large deep learning models rely on data augmentation to improve performance. To study the impact of data enhancement, we will consider several common enhancements here. There are two types of data augmentation in the vision computer domain. One is appearance transformation, such as sharpness, brightness, contrast, saturation, gray processing, Gaussian blur, and elasticity (22), another involves spatial geometric transformation, such as horizontal flipping, rotation, cropping, and resizing (23). Every enhancement strategy can transform data stochastically with internal parameters (e.g., rotation degree, noise level). Our model adopted strategies including random clipping, Affine, Gaussian blur, and Gaussian noise. **Figure 2** visualizes the augmentations that we study in this work.

### 2.2 ccRCC classification network

**Figure 3:**
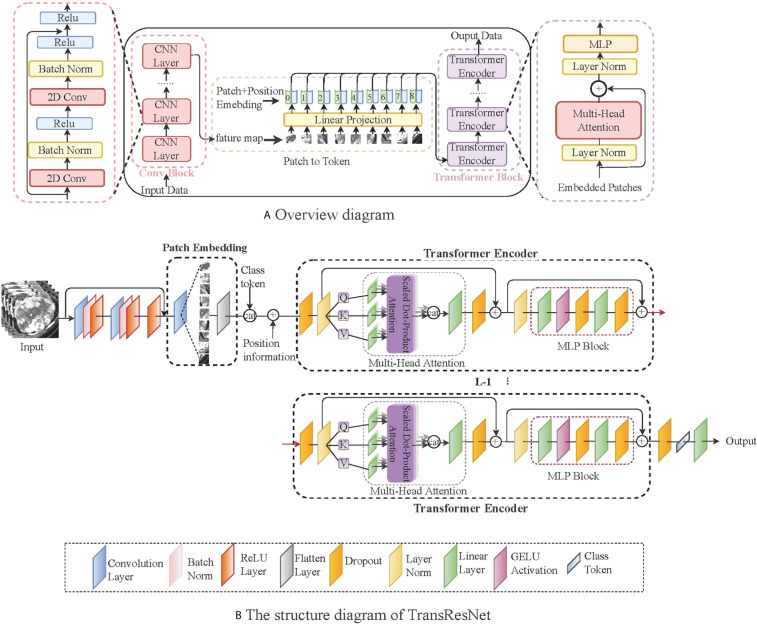
A simple network framework for TransResNet. **(A)** represents the overall architecture, mainly including the CNN structure and the transformed structure. **(B)** shows the details of TransResNet. The network framework contains 12 transformer blocks (i.e., L = 12).

To make the features extracted from the ccRCC dataset correspond to the label as much as possible, we introduced a transformer module into convolutional networks to improve performance. We illustrate the overall diagram in **Figure 3A**. Firstly, the input image is processed with several convolution blocks, and then the processed feature map is provided to the transformer block. At the end of the network, the class tokens are applied for the prediction of ccRCC. Our insights into taking advantage of convolutions and transformers are: (1) in the early network, using convolution to learn densely distributed and low-level features requires less computational cost than the transformer; and (2) in the later network, applying the transformer to learn higher-level semantic concepts and long-range dependencies in the image.

The network framework, termed TransResNet, is composed of convolution blocks, transformer blocks, and classifier (one FC layer). The convolution block adopts a pyramid structure, in which the resolution of feature mapping decreases with the increase in network depth while the number of channels increases. It consists of four phases: the first phase is a 3 × 3 convolution with stride 1 and padding 1, which is used to extract initial local features. In the last three stages, we refer to the first three layers of ResNet, in which the output channels of each layer are 16, 32, and 64, respectively. The Transformer block contains 12 repeated transformers. As shown in **Figure 3B**, each transformer consists of a multi-head self-attention (MSA) module and a multi-layer perceptron (MLP) block.

In the Transformer, we consider the input feature map *X*∈*ℛ* ^*c*×*h*×*w*^ with *c* channels and the feature shape of *h × w*. Self-attention estimates the relationship between one part of a feature map and other parts (e.g., which tumor masses are likely to come together into a complete tumor mass with maximum grade). Therefore, the feature map is divided into a sequence *X*={*x*_1_,*x*_2_,…,*x*_*n*_} ( $x\in {ℛ}^{c\times \frac{h}{n}\times \frac{w}{n}}$ ) with *n* patches. The goal of self-attention is to capture the interaction among all *n* patches by encoding each patch in terms of global contextual information. The output of the MSA layer is computed with the feature map *X* using the following equation:


(1) $$Z=MSA\left(LN\left(softmax\left(\frac{QK^{T}}{\sqrt{d_{k}}}\right)V+X_{class}+X_{position}\right)\right)+X$$

where Queries *W* ^*Q*^ , Keys *W* ^*K*^, and Value *W* ^*v*^ are learnable weights to automatically learn the importance of each patch. The input sequence *X* is projected onto these weight matrices to get *Q*=*XW* ^*Q*^ , *K*=*XW* ^*K*^ and *V*=*XW* ^*V*^ . The *d*_*k*_ is the dimension of key vector *k*, providing an appropriate normalization to make the gradient more stable. LN represents the linear normalization, *X*_*class*_ and *X*_*position*_ are randomized parameters representing classification and position information, respectively. To capture the structure of object image, position and classification information are merged into the self-attention feature map for training.

After the self-attended feature map *Z* passes through the linear layer, we put it into a MLP including two linear transformations and a GELU activation to perform the same operation on the vector of each position. The output can be obtained:


(2) Y=MLP(LN(Z))+Z

Referring to ViT (24), we take the classification information *X*_*class*_ instead of extracted image features as the input of the classifier FC to classify directly. We train our network using the cross-entropy (CE) loss between prediction and ground truth, which can be written as:


(3) $${ℒ}_{CE}=-\sum^{\;}(y\log\;\hat{y}+\left(1-y\right)\log\;(1-\hat{y}))$$

where the *y* is the real label and $\hat{y}$ is the predicted probability. The loss function represents the difference between the real label and the predicted probability.

We constructed TransResNet with three residual blocks and 12 transformer blocks. The major hyper-parameters are as follows: the optimizer is Stochastic Gradient Descent (SGD) with an initial learning rate of 0.01, momentum of 0.9, and weight decay of 5e−4. The batch size is 100 per worker. For the epochs, the learning rate is scaled linearly from 0.01 to 0.00001, and then it is divided by 10 at epochs 50, 100, and 150. The proposed model is implemented using Pytorch 1.0.1. We ran the experiments on an Ubuntu 16.04.3 server with four NVIDIA GeForce GTX 1080 cards. Specific details of the code can be seen at: https://github.com/yangmeiyi/ccRCC_project.git.

### 2.3 Model ensembles

To a certain degree, ensemble learning can improve prediction accuracy. To achieve a strong classifier, we train multiple different classifiers. We selected the following five networks as sub-networks of the integration model:

TransResNet: In medical, ResNet (25) is the preferred model because it is simple and efficient. The most common model, ResNet50, is ineffective on our dataset due to serious over-fitting. Finally, we decided to pair Transformer and resnet34.
TransDenseNet: DenseNet (26) found that some layers are randomly lost at each step in the training process, which can significantly improve the generalization performance of ResNet. Similarly, we chose to combine Transformer and DenseNet-121 with the smallest model parameters as our sub-network.
TransInception: Compared with the structure of ResNet, Inception (27) not only increases the width of the network but also embeds features of different scales. Similarly, Inception-V3 was selected by us.
TransSENet: Different from the previously mentioned networks, improving the performance through spatial latitude, SENet (28) establishes the interdependence between feature channels.
TransRegNet: RegNet (29) proposes a new design paradigm that estimates the overall network design space (depth and width) to obtain the best design. Similarly, we chose RegNetY-200MF with the minor model parameters.

We combine them through the average method. These classifiers and TransResNet are constructed in the same way. The difference lies in the construction of convolution blocks, such as TransDenseNet uses a dense convolutional network and TransInception adopts an inception network.

Suppose we have got *N* trained different classifiers. Facing a test sample *x* (*x* ∈ test dataset), the prediction results of the integrated model are a *N*-dimensional vector {*S*_1_,*S*_2_,…,*S*_*n*_} . The final score using averaging is formulated as follows:


(4) $$Final\_score=\frac{\sum_{i=1}^{N}S_{i}}{N}$$

## 3 Results

### 3.1 Clinical characteristics

This study included 759 patients, which comprised 477 low-grade [grade I (n = 25, 5.24%) and II (n = 452, 94.76%)] ccRCC patients and 282 high-grade [grade III (n = 232, 82.27%) and IV (n = 50, 17.73%)] ccRCC patients. Male and female patients are equally represented, accounting for 64% and 46%, respectively. The patient characteristics of the training and testing cohorts are shown in **Table 1**.

**Table 1:** The demographic and clinical statistics of patients with ccRCC.

Attribute	Training cohort	Testing cohort
Age (years)	56 ± 12 (589)	54 ± 11 (170)
Male	374	112
Female	215	58
Grade I	21	4
Grade II	371	81
Grade III	165	67
Grade IV	32	18

### 3.2 Performance of proposed algorithm

To validate this model, we choose Accuracy (ACC), Area Under Curve (AUC), Sensitivity (SE), and Specificity (SP) as evaluation criteria.

**Figure 4:**
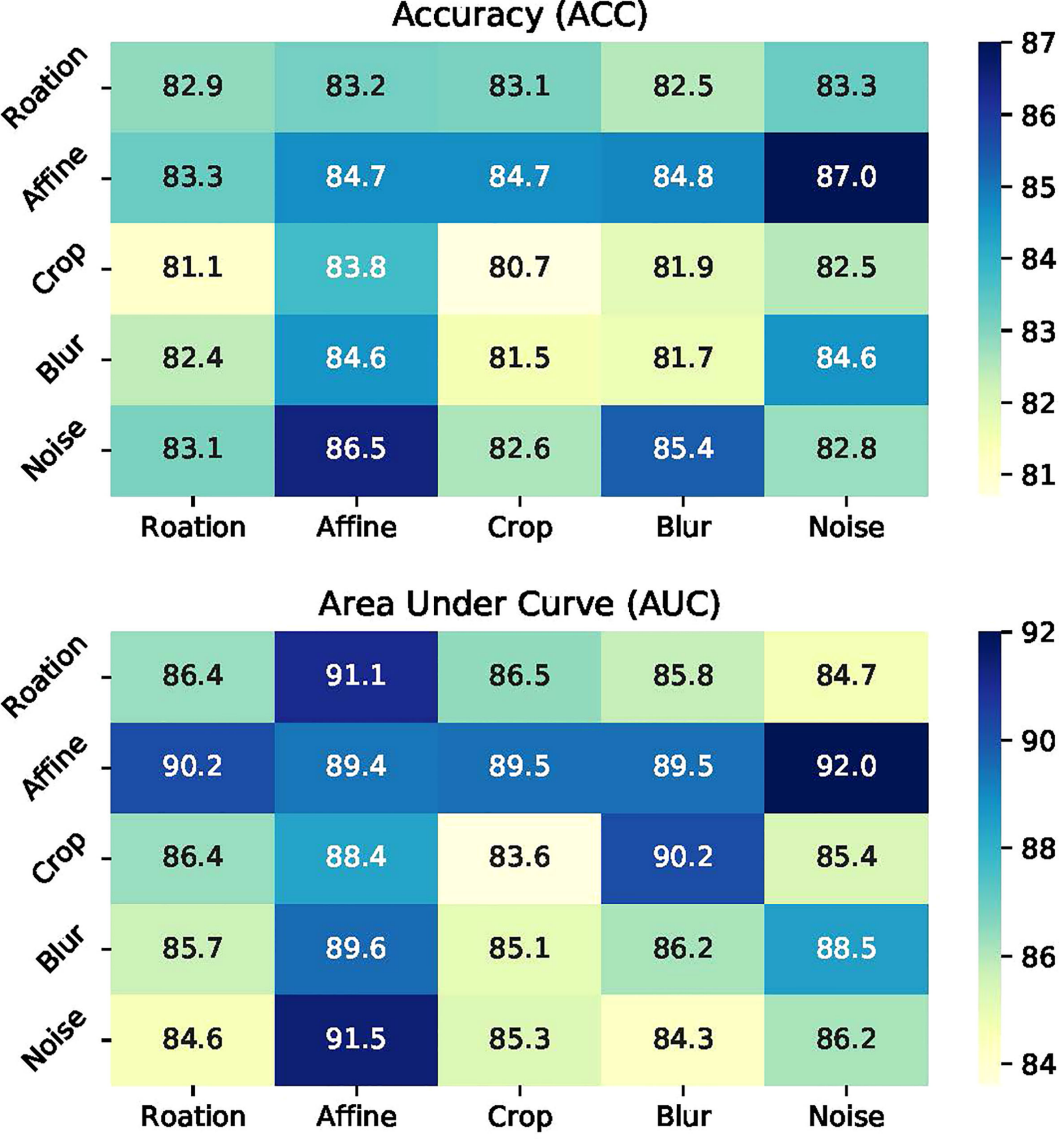
Illustrations of data augmentation operators.

**Performance with data enhancement:** To comprehend the role of enhancement strategy in detail, the individual and different combinations of them are discussed. With inconsistent images of the ccRCC, cropping as the basic data processing is applied. **Figure 4** shows the evaluation results under single and combined transformations. Even though Affine is a very effective enhancement for the model, any single transformation is insufficient for representation learning. One augmentation composition stands out: random Affine and random Gaussian noise. The best combination is more than two enhancements, such as random Rotate, random Affine, and random Crop, making the model obtain 87% ACC and 91% AUC.

**Performance on different architectures.** This section shows the effect of the transformer on ResNet (25), DenseNet (26), Inception (27), SENet (28), and RegNet (29). Each model has its own unique advantages. We present the ACC, AUC, SE, and SP of TransResNet, TransDenseNet, TransInception, TransRegNet, and TransSENet on the ccRCC dataset in **Table 2**. Each architecture was performed under the enhancement method of Crop, random Horizontal Flip, random Rotation, and random Affine.

**Table 2:** The impact of transformer on different network structures. .

Model	ACC	AUC	SE	SP
ResNet-34	82.3 ± 2.5	85.7 ± 2.3	89.4 ± 1.7	83.2 ± 1.2
TransResNet	87.1 ± 2.3	90.3 ± 2.5	91.3 ± 1.4	85.3 ± 1.5
DenseNet-121	81.5 ± 2.3	85.5 ± 2.4	80.0 ± 0.5	84.3 ± 1.2
TransDenseNet	83.9 ± 2.1	90.5 ± 2.2	80.6 ± 0.6	86.8 ± 1.0
Inception-V3	80.0 ± 2.0	83.7 ± 2.0	76.5 ± 1.2	78.8 ± 1.3
TransInception	84.3 ± 2.0	89.4 ± 2.0	83.4 ± 1.3	85.8 ± 1.6
SENet	81.8 ± 2.5	84.2 ± 2.8	76.8 ± 1.5	85.1 ± 1.4
TransSENet	85.1 ± 2.3	89.2 ± 2.5	89.1 ± 1.3	82.1 ± 1.1
RegNet	81.9 ± 2.1	84.3 ± 2.5	82.5 ± 1.5	80.0 ± 1.0
TransRegNet	82.3 ± 2.0	87.7 ± 2.5	84.7 ± 1.0	80.0 ± 0.5

We report the average accuracy and standard deviation of five time runs. ACC, AUC, SE, and SP are the accuracy, the Area Under Curve, the sensitivity and the specificity of the model on the testset, respectively.

**Figure 5:**
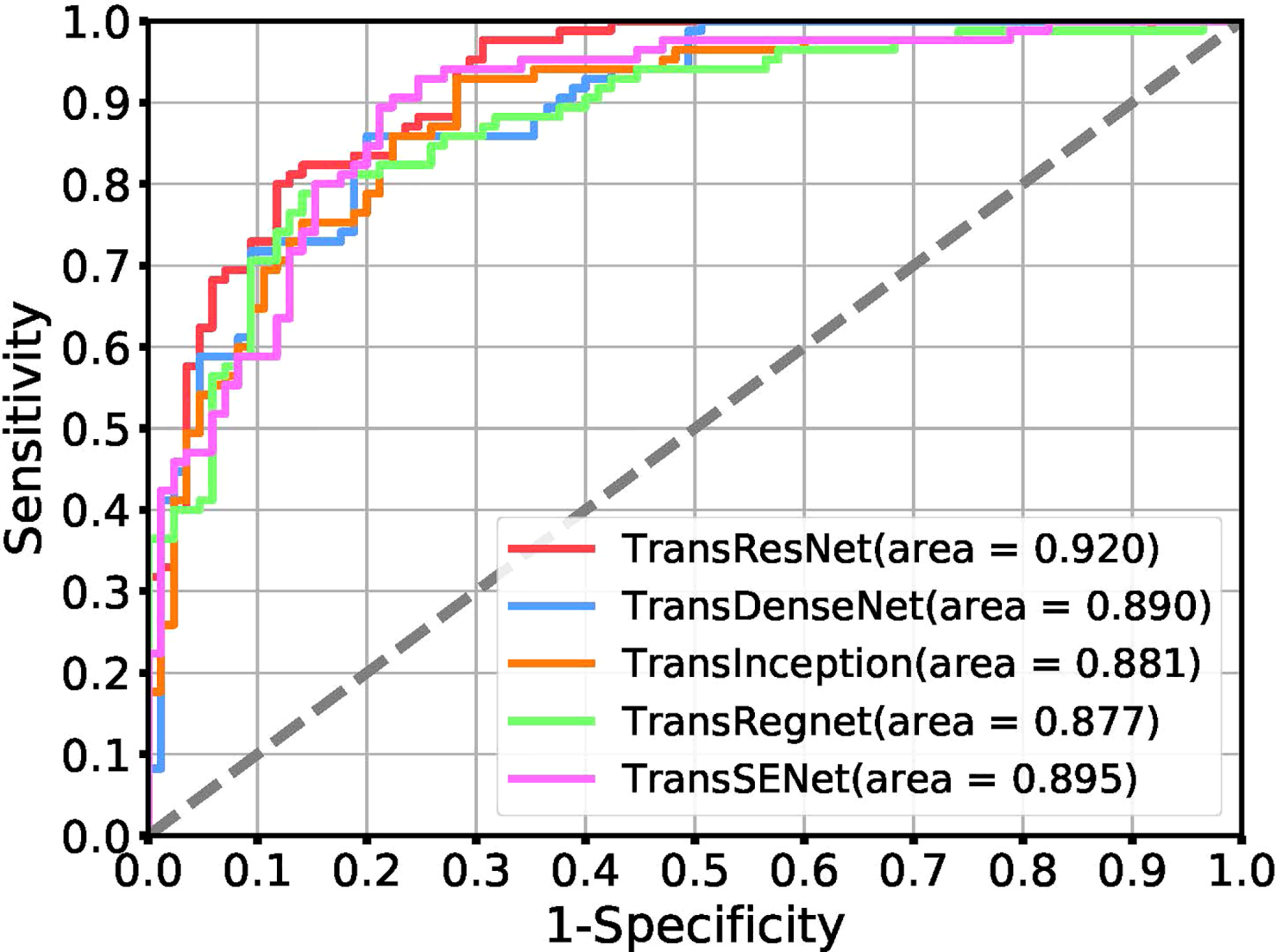
Receiver operating characteristic (ROC) curves for the task of tumor classification using a positive ratio feature.

**Table 2** shows the experimental results of different network architectures. The Receiver Operating Characteristic (ROC) curve is a metric that can provide a pure index of accuracy, which has been accepted by every researcher and applied in medical studies. We show the ROC curve in **Figure 5**.

**Ensemble results:** Ensemble learning is a popular way to improve robustness and accuracy by training a group of heterogeneous models. These heterogeneous models are combined by different strategies such as voting, averaging, stacking, and blending. This study analyzes various ensemble strategies, and the results are shown in **Table 3**. The integrated model consists of a single model: TransResNet, TransDenseNet, TransInception, TransRegNet, and TransSENet.

**Table 3:** Ensemble results under different strategies.

Model	ACC	AUC	SE	SP
TransResNet	85.8	90.3	91.3	85.3
TransDenseNet	83.9	90.5	80.6	86.8
TransInception	84.3	89.4	83.4	85.8
TransRegNet	82.3	87.7	84.7	80.0
TransSENet	85.1	89.2	89.1	82.1
Ensemble(voting)	85.8	90.5	91.4	89.8
Ensemble(averaging)	**86.5**	**91.2**	**92.1**	**89.5**
Ensemble(stacking)	86.1	90.8	89.1	91.1
Ensemble(blending)	86.1	90.0	90.0	85.5

ACC, AUC, SE, and SP are the accuracy, the Area Under Curve, the sensitivity and the specificity of the model on the testset, respectively. The averaging strategy of ensemble shows the best results (the seventh column, highlighted in bold).

### 3.3 Comparison with SOTA methods

Does our proposed solution lead to better performance than pure transformers? To answer this question, we compared pure transformer networks ViT (24) and CaiT (30) with the smallest parameters and a hybrid network Conformer (31) trained in parallel by convolution and transformer in **Table 4**. In the training process, ViT-Small and CaiT-Small are suitable for our dataset, but the results are still unsatisfactory. In particular, the hybrid structure Conformer with huge model parameters is difficult to train on small dataset ccRCC, resulting in the worst performance, although its classification results on ImageNet are better than ViT and CaiT.

**Table 4:** Comparison with the state-of-the-art transformer.

Model	ACC	AUC	SE	SP
TransResNet	**85.8**	**90.3**	**91.3**	**85.7**
ViT-Small	78.6	83.5	88.5	81.3
CaiT-Small	79.4	83.2	89.1	82.0
Conformer	76.4	79.3	83.5	65.7

ACC, AUC, SE, and SP are the accuracy, the Area Under Curve, the sensitivity and the specificity of the model on the testset, respectively. Our model TransResNet obtains the best results (the first column, highlighted in bold).

**Figure 6:**
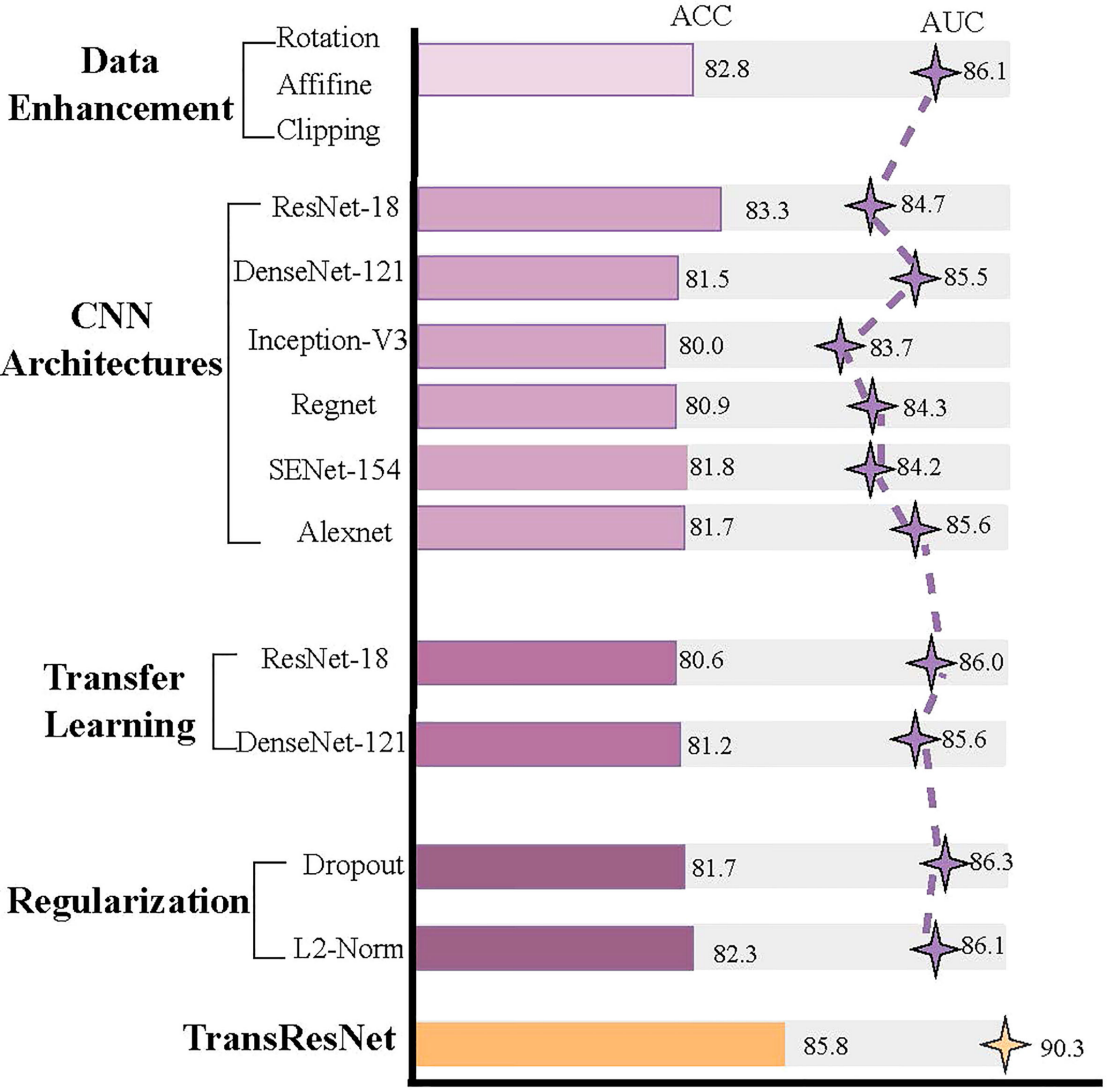
A series of experimental results about CNN.

Does the transformer improve performance compared to CNN? For CNN, we have done a series of experiments with ccRCC for comparison. The experiments included Data Enhancement, CNN architecture, Transfer Learning, and the Regularization Method. The experimental results are shown in **Figure 6**. In the experiment of data enhancement, the performance of the CNN model has definite improvements after random Affine. However, other data enhancement methods are not effective for the dataset. We found that the CNN architectures have little effect on the Fuhrman Grade of ccRCC, although the advantages of each structure are different. From various experimental results, the model based on CNN is difficult to break through the bottleneck of AUC (e.g., 85 AUC). Our method, TransResNet, easily surpasses CNN algorithms using various tricks in ACC and AUC.

### 3.4 Comparison with transfer learning

Transfer learning is widely used in medical image processing, such as type 2 diabetes (32), 3D genome folding (33), and papillary thyroid carcinoma (34), etc. Many factors make it impossible to establish large-scale datasets such as ImageNet in the medical field, so the limited data restricts the performance of deep neural networks. Some studies reckon that the pre-trained model obtained on ImageNet can be fine-tuned on the medical dataset to acquire high performance. The experiment by Shin et al. proved that although there are differences between natural images and CT images, CNN fully trained on large-scale well-annotated datasets may still be transmitted to make the medical image recognition task more effective (35). Therefore, we compared the performance of our method and transfer learning on ccRCC. We transferred the ResNet, DenseNet, and ViT trained with ImageNet to ccRCC for fine-tuning, respectively. In transfer learning, to make the network training more thorough, we follow the method of (35): all CNN layers are fine-tuned with a learning rate 10 times lower than the default learning rate, except for the last layer. The last fully connected layer is randomly initialized and newly trained to adapt to the new object categories in the ccRCC application. Its learning rate remains at the original 0.01. The results are shown in **Table 5**.

**Table 5:** The comparison results of our method and transfer learning.

Transfer Model	ACC	AUC	SE	SP
ResNet18	80.6	86.0	83.3	78.8
ResNet34	80.2	85.6	86.3	78.2
DenseNet121	81.2	85.6	78.9	89.4
TransResNet	**85.8**	**90.3**	**91.3**	**85.3**
ViT-tiny	74.7	75.6	64.7	84.7

The comparison models are ResNet, DenseNet, and ViT-tiny parameterized by pre-training using the ImageNet dataset. Our model is trained from random initialization.

ACC ,AUC ,SE, and SP are the accuracy, the Area Under Curve, the sensitivity and the specificity of the model on the testset, respectively. Our model TransResNet obtains the best results (the seventh column, highlighted in bold).

In the experiment, we found that although transfer learning can speed up convergence early in training, it does not improve final accuracy. As shown in **Table 5**, the accuracy of our proposed method can quickly catch up with the best of transfer learning and stay higher.

### 3.5 Analysis of noise labels

**Figure 7:**
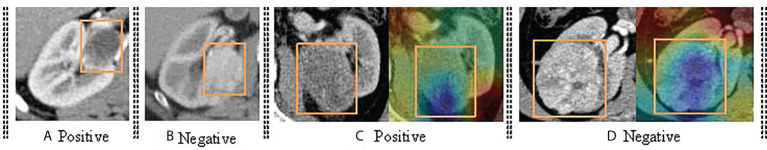
The demonstration of error classification. It mainly includes four categories. **(A)** shows the the CT image of positive samples; **(B)** shows the the CT image of negative samples; **(C)** shows the CT image of positive samples and corresponding class activation map; **(D)** shows the CT image of negative samples and corresponding class activation map.

Previous articles (15, 17) studied the impact of CNN-based deep learning on ccRCC. These studies prove that the features from CT images extracted by CNN can be effectively used for ccRCC grading and obtaining the SOTA performance. Due to the inconsistency between extracted features and labels, some data are always misclassified by the CNN-based algorithm. For example, 21 CT images in our test set could not be classified correctly, including three low-grade images and 18 high-grade images. This phenomenon is consistent with the fact that high-grade images contain more noise information. In other words, the features extracted from tumor images scanned by high-grade ccRCC CT scans are not always related to Fuhrman nucleus grades III and IV, and some of the features are related to Foreman nucleus grades I and II. **Figure 7** shows the case of misclassification data, in which **Figure 7A** is a positive sample, but it is classified as a negative example. **Figure 7B** is a negative example sample of classification error. **Figure 7C** is a class activation map of a positive example sample of classification error. **Figure 7D** is a class activation map of a positive example sample of classification error. We found that: (1) similar CT images with different categories are difficult to distinguish (e.g., **Figures 7A, B**), and (2) some CT images are not activated correctly (e.g., **Figures 7C, D**).

**Figure 8:**
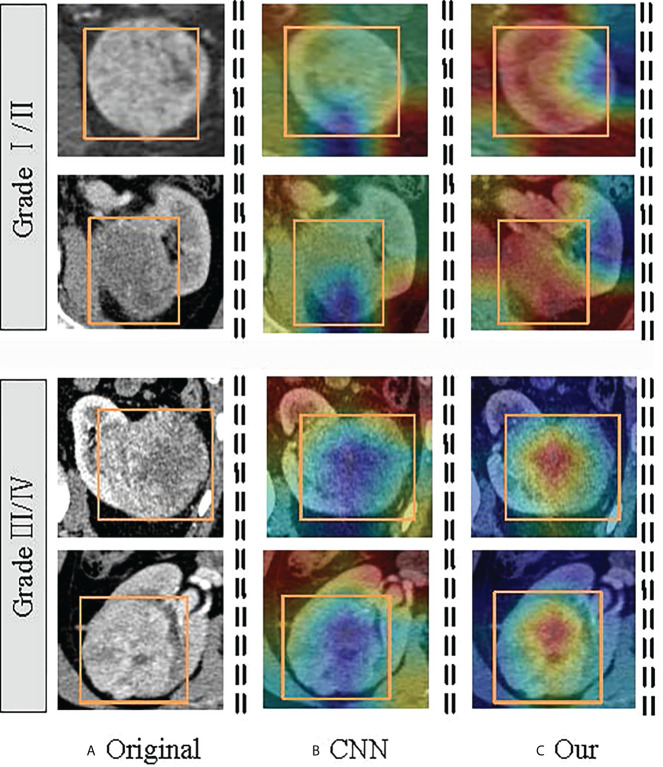
Visualization of the class activation map generated by the last transformer layer on images from the ccRCC. The yellow box indicates the lesion area. The color red denotes higher attention values, and the color blue denotes lower. **(A)** is the original image; **(B)** shows the class activation map of the CNN model; and **(C)** is the class activation map of the TransResNet model.

The Transformer offers many advantages over traditional CNN algorithms for ccRCC grading. For example, Transformer can capture the representation of global images and establish long-distance relationships in CT images, reflecting the microscopic heterogeneity changes of tumors more comprehensively and providing a more accurate diagnosis than all CNN algorithms. In addition, the complementary strengths between CNN and Transformer can be adapted to the requirements of specific clinical environments. After the optimized classifier TransRsenet, 14 CT images in our test set could not be correctly classified, including two low-grade images and 12 high-grade CT images. **Figure 8** shows the class activation map of these error data to show the shift of attention of the model during training. Compared with the convolutional network, the model with the proposed method makes objects clearer and more accurate than the original ones.

In addition to the comparison of convolution algorithms, we also compared the network architecture of SOTA, such as the transformer-based ViT, CaiT, and Conformer, obtaining the best grading results under ACC, SE, SP, and AUC. In conclusion, we demonstrated the effectiveness of the Transformer module over ccRCC grading. Furthermore, the combination of CNN and Transformer mitigates the noise label problem in ccrRCC.

### 3.6 External validation

We evaluate our model based on one external validation dataset, the Cancer Genome Atlas-Kidney Renal Clear Cell Carcinoma (TCGA-KIRC) (36, 37). The TCGA-KIRC focused on connecting cancer phenotypes to genotypes by providing clinical images matched to subjects from The Cancer Genome Atlas (TCGA). Clinical, genetic, and pathological data resides in the Genomic Data Commons (GDC) Data Portal, while the radiological data is stored in The Cancer Imaging Archive (TCIA). Here we just use the CT radiological data. We selected 20 patients (e.g., 10 low-grade samples and 10 high-grade samples) from 227 patients as our external validation data. The results of our model TransResNet on the data are: 81.9% ACC, 85.4% AUC, 76.6% SE, and 87.2% SP.

## 4 Discussion

This retrospective study comprehensively investigates the pros and cons of different deep learning models based on two-phase CT images for discriminating low- and high-grade ccRCC. According to data scarcity and noise labels, a transformer-based deep learning model, TransResNet, is proposed. After a thorough comparison, the proposed discriminative model, TransResNet, achieved satisfactory performance. In addition, we find that this model can effectively alleviate the impact of noise labels.

At present, the analysis of radiomics is a common paradigm to predict the ccRCC. Previous studies analyzed the texture of non-enhanced CT through machine learning to obtain universal features for higher accuracy. For example, Coy et al. have shown that high-grade ccRCC lesions are significantly larger and have more calcifications, necrosis, aggregation system infiltration, and unclear tumor margins than low-grade ccRCC lesions (38). In addition, several studies (39, 40) have proved that high-grade tumors tend to be larger than low-grade. However, our experiment found high-grade lesions are not significantly different from low-grade CT images. This is consistent with the findings of (12) and (13). The performance of image-based quantitative indicators varies on different datasets, and their adequacy needs further verification. In this study, we use deep learning to analyze the CT images, which can automatically discover pixel-level features, supporting a more powerful model.

In ccRCC, some high-grade lesions have low-grade features due to unavoidable sampling bias. It was also interesting to find that convolution-based networks focus on local pixel subsets, which results in a tendency to learn local patterns so that the relationships within images are ignored. As a result, in the complex samples, the convolution kernel cannot effectively make decisions, which leads to unsatisfactory grading accuracy. CNNs uniformly process CT images regardless of their importance. In contrast, the transformer uses a self-attention mechanism instead of a CNN, which can establish long-range dependencies in images. The Transformer has been widely used for medical tasks with high accuracy, e.g., prostatic segmentation (41), delineating the epicardium and endocardium (42), multi-modal medical image classification (18), etc. The Transformer can not only be used for segmentation but also carries an advantage in processing images with noisy labels due to its way of capturing image features. However, transformer networks require a large-scale dataset for training because the transformer lacks inductive bias (24). Thus, scarce medical data leads to severe over-fitting, which may further reduce the reliability of the classifier. Therefore, we take advantage of the CNN and a transformer to extract the features of CT images. The introduced transformer module can help CNN obtain the relationship between the pixel blocks inside the image. We find that this hybrid classifier is superior to a single classifier, with ACC increasing from 83% to 87% and AUC increasing from 85% to 90%.

Correctly grading complex samples is one of the significant indicators for evaluating the strength and stability of a deep learning model, which reflects the ability to extract general features. Our method shows some advantages when faced with noise samples and difficultly graded examples (see *Analysis of noise labels* section). As is known to all, different models have respective preferences for data, and the features learned from a single model are not necessarily reliable. This paper trains a set of heterogeneous models to improve robustness and accuracy by integrating their extracted features, with ACC increasing from 85.8% to 86.5% and AUC increasing from 90% to 91.2%. This study shows that our method can effectively reduce the difficulty of grading caused by noise labels. This method can be widely applied to other medical grading tasks, e.g., the grade of neurogliomas and meningiomas.

Our study has several limitations. First, Fan et al. (12) found that the classifier based on a three-phase CT image is better than that based on a single-phase CT image. Theoretically, non-enhanced CT images can provide additional information for diagnosis. However, this study only used the data from the arterial and portal vein phases due to fewer non-enhanced CT images and inapparent texture information. Additionally, we did not collect additional validation sets because the original DICOM data of the ccRCC are difficult to obtain. However, the superiority of the transformer in the feature extraction can be reflected in the training and testing.

## 5 Conclusion

This paper studies the application of transformer architecture to ccRCC classification. We first collected a high-quality ccRCC CT scan dataset containing more than 759 patients with pathological proven. Then, a hybrid structure, termed TranResNet, is proposed, which compromises the merits of CNN and Transformer. Unlike other transformer models, TranResNet does not require pre-training on large-scale datasets. Finally, we conducted extensive experiments on ccRCC datasets to verify our method. TransResNet achieves good performance over ConvNets and other related transformer architectures, demonstrating promising results in ccRCC classification. We hope it will help future research on this subject, and it can cooperate with radiologists to classify the ccRCC in an actual clinical situation.

## Data availability statement

The original contributions presented in the study are included in the article material, further inquiries can be directed to the corresponding author.

## Ethics statement

This diagnostic and observational study was approved by the institutional review board (West China Hospital, Sichuan University). Written informed consent for participation was not required for this study in accordance with the national legislation and the institutional requirements.

## Author contributions

MY designed the study and all laboratory studies, performed experiments, analyzed the data, and wrote the manuscript. ML, JD, and XC performed laboratory experiments. XH, YW, QL, and SW collected and analyzed the ccRCC data. LX and FZ provided help in data and statistical analysis. LW and XW participated in data analysis and interpretation. ML and BS oversaw the design of the study and all laboratory studies, data analysis, and interpretation, and wrote the manuscript. All authors contributed to the article and approved the submitted version.

## Funding

This work was supported by the Medico-Engineering Cooperation Funds from University of Electronic Science and Technology of China (No. ZYGX2021YGLH213, No. ZYGX2022YGRH016), the Municipal Government of Quzhou(Grant 2021D007, Grant 2021D008, Grant 2021D015, Grant 2021D018), as well as the Zhejiang Provincial Natural Science Foundation of China under Grant No. LGF22G010009.

## Conflict of interest

The authors declare that the research was conducted in the absence of any commercial or financial relationships that could be construed as a potential conflict of interest.

## Publisher’s note

All claims expressed in this article are solely those of the authors and do not necessarily represent those of their affiliated organizations, or those of the publisher, the editors and the reviewers. Any product that may be evaluated in this article, or claim that may be made by its manufacturer, is not guaranteed or endorsed by the publisher.
